# Integration of an Aerosol-Assisted Deposition Technique for the Deposition of Functional Biomaterials Applied to the Fabrication of Miniaturised Ion Sensors

**DOI:** 10.3390/nano11040938

**Published:** 2021-04-07

**Authors:** Antonio Ruiz-Gonzalez, Kwang-Leong Choy

**Affiliations:** Institute for Materials Discovery, Faculty of Mathematical & Physical Sciences, University College London, 107 Roberts Building, Malet Place, London WC1E 7JE, UK; a.gonzalez.16@ucl.ac.uk

**Keywords:** ion-selective electrodes, PVC biomaterials, Nernst sensitivity, potassium ion sensor

## Abstract

Ion-selective electrodes are at the forefront of research nowadays, with applications in healthcare, agriculture and water quality analysis among others. Despite multiple attempts of miniaturization of these polyvinyl chloride (PVC) gel-based ion sensors, no ion-sensing devices with a thickness below the micrometer range, and operating using open circuit potential, have been developed so far. This work reports the causes of this thickness limitation in potassium-selective sensors. Highly homogeneous ion-sensing films were fabricated by a method based on aerosol assisted chemical vapour deposition, leading to smooth surfaces with 27 ± 11 nm of roughness. Such homogeneity allowed the systematic study of the performance and ionic diffusion properties of the sensing films at sub-micrometer scales. Sensitivities below the Nernst response were found at low thicknesses. The nature of this reduction in sensitivity was studied, and a difference in the superficial and bulk compositions of the films was measured. An optimal configuration was found at 15 µm, with a good selectivity against Na^+^ (K_K_^+^_, Na_^+^ = −1.8) a limit of detection in the range of 10^−4^ M and esponse time below 40 s. The stability of sensors was improved by the deposition of protective layers, which expanded the lifespan of the ion sensors up to 5 weeks while preserving the Nernst sensitivity.

## 1. Introduction

Ion-selective electrodes (ISE) are crucial in a wide range of fields including agriculture and crop production [1], diagnosis, used for the monitoring of multiple chronic conditions such as chronic kidney disease, diabetes or cardiovascular diseases among others [2,3,4], or industrial process such as fermentation [5]. To date, a plasticized polymeric film, commonly containing polyvinyl chloride (PVC), where one or more ionophores are embedded represents the preferred option for the fabrication of ion sensors [6]. The performance of these ion sensors is related to the ionic exchange capabilities of the sensing films, and the electrochemical potential of the working electrodes increase when they are subjected to an electrolyte solution, due to the diffusion of electrolytes inside the polymeric films [7]. Ionophores, which are specific chelating molecules with affinity towards certain electrolytes are commonly used to favor the ionic exchange of the target analyte and bring selectivity to the ion detection.

The employed polymer and plasticizer play a pivotal role in the performance of ion sensors due to their effects on the ion diffusion and dielectric properties of the films. PVC is a common biomaterial with good biocompatibility. However, this polymer has been shown to degrade over time, generating oxygen radicals and chloride ions [8]. Despite current attempts to replace the PVC polymer with other compounds, such as polyurethane or silicone rubber, PVC remains as the preferred choice given its significantly lower equilibration time. Such equilibration time is in the range of 10–20 h in the case of PVC films [9], and can reach up to months or years when polymers with lower ion permeability are employed [10,11]. In addition, as mentioned, PVC is a widely used polymeric biomaterial [12], which has been used for the manufacturing of multiple medical devices such as blood bags, infusion devices, catheters and respiratory masks [13]. As such, PVC remains as the preferred option for the development of ion-selective electrodes, with a proven performance when used as ion sensor [6,14]. Thus, currently there is a high interest in the development of fabrication methods that can be applied to the deposition of PVC-based films and coatings, with potential applications in healthcare and ion monitoring among others.

Despite the high shelf stability of the PVC [15], the lifetime of PVC-based ion sensors is limited due to a sustained exudation of the employed plasticizer over time, which decreases the sensitivity of the films and compromises its long-term performance [15,16]. The employed plasticizer in the polymeric sensing films has an impact on the selectivity of the sensing films since they can change the kinetics of ion diffusion and permselectivity [17]. The development of devices for the detection of magnesium and calcium ions represent an example of how the film composition can change the selectivity of the ion sensors. The use of high dielectric constant plasticizers such as o-nitrophenyl°Ctyl, ether, improves the selectivity of Ca^2+^-selective sensors by reducing the energy transfer of divalent ions. Consequently, the selectivity of calcium-sensing devices is improved compared to monovalent ions [18,19,20].

The mechanism of response of the polymer film-based ion sensors was reported by Ishimatsu et al. [21]. This response is a consequence of the ionic transfer of the electrolytes from the sample phase to the sensing films, and chelation with the ionophores. From a theoretical point of view, the electrochemical performance of ion sensors is described by the Eisenmann–Nikolskij Equation (1):

(1) $$E_{out}=E_{i}^{0}+\frac{2.303\times R\times T}{z_{i}\times F}\times \ln(a_{i}+\sum_{j}k_{i,j}\times a_{j}^{\frac{z_{i}}{z_{j}}})$$

where, *E_out_* is the electrochemical potential, *K_i,j_* represents the selectivity coefficient, *z_j_* and *z_i_* is the charge number of the interference and target ions, R is the universal gas constant, *T* is the temperature, *F* is the Faraday constant, $E_{i}^{0}$ is the standard potential, *a_i_* is the activity of the primary ions, and *a_j_* the activity of secondary ions. This formula relates the activity of ions in solution with the measured potential at the working electrode. A consequence of this equation is the thermodynamical limit in sensitivity of ion sensors operating under equilibrium conditions of 59 mV Log[C]^−1^ at room temperature [22]. This value is normally described as “Nernst sensitivity”, since it is the standard sensitivity achieved by ion-selective electrodes as predicted by the Nernst equations. On the contrary, lower sensitivities are described as “sub-Nernst sensitivity”, which take place in films with a suboptimal composition. However, in principle, such sensitivity is not limited by the thickness of the films, and only the selectivity towards the different ions and the activity of such ions can change the sensitivity of the films. Thus, reducing the membrane thickness could be beneficial to reduce the time-response of ion sensors by minimizing the diffusion paths of electrolytes while providing a Nernst sensitivity. This miniaturization could offer a true monitoring of electrolytes.

Traditionally, relatively thick PVC membranes, being in the range of 100–200 μm are used in the literature for the fabrication of all-solid-state ion-selective electrodes [22,23]. While there is broad agreement in the need for miniaturized sensing devices for the real-time monitoring of electrolytes in biological solutions, to the best of our knowledge, no solid-state sensors with a thickness in the sub-micrometer range, and operating at zero-current open circuit potential have been reported. In addition, nowadays there is an unmet need for the development of low-cost fabrication techniques to allow the mass manufacturing of sensing devices at sub-micron scales with good reproducibility and well-controlled dimensions.

The deposition of ion-selective films has traditionally been performed by drop casting. Although this technique has been proved to be a cost-effective method for thick film sensors (in the range of 100 µm), it leads to a non-homogeneous deposition of membranes and poor control over its thickness.

Screen-printed electrodes have been developed for a variety of ionic species such as citalobram hydrobromide [24], naphazoline hydrochloride [25], and copper [26]. Although this technique represents an improvement in terms of material homogeneity after deposition and scale-up production, it shows some limitations due to the amount of material required and the time required for the deposition. The use of spin coating represents a step forward in this field, allowing the fabrication of thin films with well-controlled thicknesses. This method was applied by Kabagambe et al. [27] for the fabrication of 1 µm thick films that could measure calcium ions by using stripping voltammetry. However, this fabrication method is unsuitable for mass-scale production since it is limited by the substrate shape and, in most cases, to only one sample per batch [28]. In addition, the material efficiency of the spin coating method is low, with 95–98% of the employed material being flung off [29], which could represent a burden when relatively expensive molecules such as ionophores are needed, greatly increasing the production costs.

Aerosol-assisted chemical vapor deposition (AACVD) represents a promising alternative for the fabrication of ISE with well-defined surface morphologies and high control over the films dimensions. AACVD is a variant of chemical vapor deposition in which the chemical precursors are aerosolized into sub-micrometer liquid droplets in a gas medium by using an ultrasonic aerosol generator. The chemical precursor is prepared by dissolving the material in a suitable solvent and an aerosol is generated. The aerosol is delivered onto a heated substrate, evaporating the solvent on contact. This technique has several advantages over other CVD based methods due to its operational simplicity, reduced cost and it does not need vacuum [30].

Within the present work, a fabrication technique namely aerosol assisted chemical deposition (AACD) was adapted for the deposition of highly homogeneous PVC-based ion sensing films, with a roughness in the nanometer range. This technique is based on the standard AACVD method reported by Choy [30], and allowed a fine control over the thickness of the films, enabling the manufacturing of up to 100 nm thick sensors with a roughness down to 27 nm. As such, it could be employed for the development of miniaturized devices and coatings with well-defined dimensions. This technique could also be applied to multiple polymers and different molecular weights, demonstrating the versatility of the system for the deposition of a wide range of functional films. The method described here could additionally overcome the limitations of current deposition methods applied for the fabrication of thin sensing films. Contrary to spin coating, this fabrication technique could be used for the deposition of multiple electrodes simultaneously, and it was not limited by the substrate morphology. Thus, AACD combined the possibility of reducing the film dimensions combined with a low roughness level and is suitable for the low-cost and non-vacuum mass manufacturing of ion sensors with well-controlled morphologies. This surface homogeneity allowed the systematic study of the effects of thickness on the sensitivity and time-response of the devices at sub-micron scale for the first time. The novel deposition technique was additionally applied for the fabrication of a biocompatible protective film, which could expand the lifespan of the thin film-based sensing devices for at least 5 weeks after a continuous exposure to an aqueous solution containing electrolytes. Thus, the novel method for the deposition of PVC films reported here shows a plethora of potential applications in ion monitoring and healthcare among others.

## 2. Materials and Methods

### 2.1. Materials

All the reagents were purchased form Sigma Aldrich, unless indicated; high molecular weight polyvinyl chloride (84 kDa), medium molecular weight PVC (79 kDa), low molecular weight PVC (49 kDa), cyclohexanone, bis(2-ethylhexyl) sebacate, tetraphenyl borate, calcium chloride, and potassium chloride. Valinomycin was purchased from MP Biochemicals, Santa Ana, CA, USA. Simulated body fluids were purchased from Xi’an Hat Biotechnology Co., Ltd, Xi’an (China).

### 2.2. Device Fabrication

**Figure 1:**
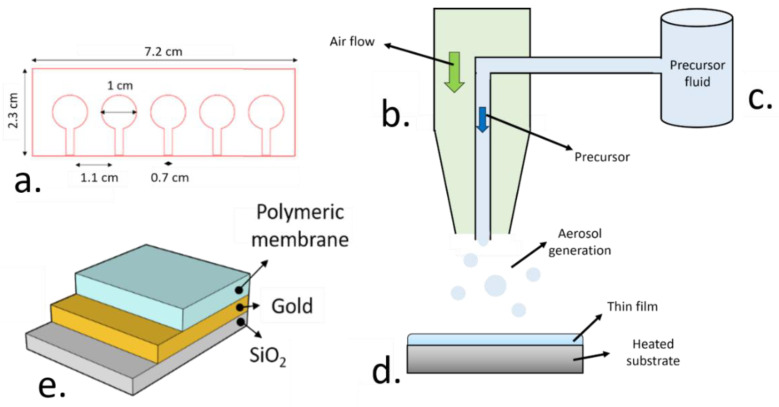
(**a**). Schematic representation of the methacrylate mask employed for the deposition of the sensitive polymer films. (**b**). Representation of the aerosol-assisted chemical deposition (AACD) process, including a pneumatic atomizer nozzle, where the aerosol is generated by using an air flow. (**c**). Such precursor solution is stored in a reservoir at atmospheric pressure. (**d**). The thin gold electrode films generated by plasma sputtering using the methacrylate mask are used as substrate for the deposition of the polymeric sensing films. (**e**). The final device consisted of the silica glass substrate where the thin gold electrodes were deposited along with the plasticized polymeric sensing film.

Firstly, a 50 nm thick gold electrode film was deposited onto a silica glass slide by using plasma sputtering (Q150RES, Quorum technologies, Lewes, UK). A custom-made methacrylate mask, patterned by using laser cutting (PLS6.75, Universal Laser Systems, Wien, Austria) was used for the deposition of these gold electrodes, which presented a diameter of 1 cm (Figure 1a). A PVC-based sensing film was then deposited on top of these gold electrodes.

Using cyclohexanone as a carrier fluid, with a standard composition of 38% PVC, 58% 2-(ethylhexyl) sebacate (DOS), 2% valinomycin and 2 wt.% tetraphenyl borate (TFB), ion-sensitive membranes were deposited by aerosol assisted chemical deposition (AACD). The precursor solution was stored in a reservoir under atmospheric pressure, and an aerosol was generated by the venturi effect using a pneumatic nebulizer [31]. The generated aerosol was focused towards the thin electrode film, which was heated at different temperatures (20–60 °C, Figure 1e). Up to 5 different electrodes could be fabricated simultaneously.

### 2.3. Thermal Characterization of the Plasticized Polymer Films

The effects of temperature on the properties of the polymer sensing films during the fabrication were determined. Thin plasticized PVC membranes were deposited onto a gold electrode by AACD. A differential scanning calorimeter (DSC, N5370212, Perkin Elmer, Waltham, MA, USA) analysis was conducted to confirm the chemical composition and thermal stability of the pure PVC films, plasticized PVC films using DOS and valinomycin by determining the boiling point and thermal decomposition. As such, this study allowed the determination of the working range for the fabrication process, since it allowed the study of degradative processes taking place in the polymer films during the polymer deposition at high temperatures. After the fabrication temperature was optimized, this fabrication process was further optimized by studying the effects of deposition time on the films. The roughness and thickness of the films was determined on each case using stylus profilometer (Dektakxt, Bruker, UK). 

### 2.4. Study of the Sensitivity of the Sensing Films

An electrochemical characterization of the final device containing the polymeric film and gold electrodes was conducted by monitoring the open circuit potential (OCP) of the electrodes within a range of electrolyte concentrations using an electrochemical station (Metrohm, Autolab BV, The Netherlands) on a three-electrode cell. A platinum counter electrode and an Ag/AgCl reference electrode were used, and the sensors were tested at different concentrations of KCl: 10^−6^, 10^−5^, 10^−4^, 10^−3^, 1.6 × 10^−3^, 4 × 10^−3^, 10^−2^ and 1.6 × 10^−2^ M. All the sensors were conditioned previously by incubating them in a 0.1 M solution of KCl overnight, and different polymer thicknesses were tested by depositing the sensing films using AACD. The limit of detection of the potassium-sensing films was additionally established. In this case, the electrochemical potential of the sensing films was first recorded in deionised (DI) water for 5 min. The intersection between this value and the calibration curve obtained after subjecting the potassium-selective sensors to different concentrations of KCl was then calculated, which indicated the limit of detection.

The determination of the selectivity of the films was carried out by using the fixed interference method. In this case, the films were initially subjected to a concentration of 0.1 M NaCl, and the same concentrations of KCl used during the calibrations were employed. The intersection between the initial calibration plot and the calibration with NaCl was then used for the study of the selectivity of the films.

### 2.5. Determination of the Ion Intake by Quartz Microbalance and Scanning Electron Microscopy (SEM) Morphology

The use of a quartz microbalance allowed the characterization of the mass variations in the polymer films due to the exchange of ions with the sample solution. This test could be employed for the study of the performance of thin films at low thicknesses. A first study was conducted to determine the effects of the plasticizer in the water absorption of the polymer membrane. Films with different contents of plasticizer (10, 20, 30, 40, 50, 60, 70, 80 and 90 wt.%) were immersed in DI water for 75 min and the changes in weight of the films were measured using a quartz microbalance (Q-sense, Biolin Scientific, Västra Frölunda, Sweden). The effects of different thickness within the sub-micrometer scale were then determined by fabricating membranes using a standard composition of 1:3 PVC and DOS as plasticizer. The changes in weight of the films after equilibration in DI water for 2 h and immersion in KCl 1 M for 2 h were then measured. 

A cross-section of the polymer films was visualized by scanning electron microscopy (SEM) to determine their morphology after their fabrication. The plasticized polymer samples were first immersed in liquid Nitrogen to fix the surface structure, and they were sectioned. These samples were studied by SEM (EVO LS15, ZEISS, Jena, Germany) and the chemical composition of the cross-section was evaluated by energy-dispersive X-ray spectroscopy (EDS, Aztec, Oxford instruments, Abingdon, UK). This technique could additionally be used for the study of the degradation of the polymer films, by allowing the characterization of the composition of microparticles obtained from the samples of water in contact with the sensing films for 1 month. The composition of the water samples from the exposure of the films could be confirmed by Fourier transform infrared (FTIR) spectroscopy (L160000A Perkin Elmer, Waltham, MA, USA), which allowed the study of plasticizer exudates. Finally, the surface homogeneity of the sensing films was determined by atomic force microscopy (AFM, Cypher S, Oxford Instruments, Abingdon, UK) and the porosity of the plasticized PVC films was determined by Brunauer–Emmett–Teller pore analysis (BET) (Quantachrome, NOVATouch, Boynton Beach, FL, USA).

### 2.6. Deposition of the Upper Protective Films

After the optimization and characterization of the thin polymer films as ion sensors, a protective coating was designed to improve their long-term stability. Such protective coating consisted of a poly(dimethyl) siloxane (PDMS) and ethyl cellulose (EC) mixed film fabricated by AACD. The composition of this protective film was optimized by varying the ratio between EC and PDMS (0, 20, 40, 60, 80 and 100 wt.% of both PDMS and EC). In all cases, a both EC and PDMS were mixed up to a weight of 10 mg, and they were successively diluted in 10 mL dimethyl formamide (DMF). This precursor solution was deposited directly onto the plasticized polymer sensors. The sensors were then preconditioned overnight by using a 0.1 M KCl solution.

### 2.7. Characterization of the Protective Films

An optimization of the composition of the upper protective films was carried out in terms of roughness, hydrophobicity and protein adsorption. After the deposition of these protective coatings by AACD, the roughness of the films was measured using a stylus profilometer, and the water contact angle was determined using an optical tensiometer (Attension, Biolin Scientific, Västra Frölunda, Sweden). The adsorption of albumin onto the surface of the protective films was determined by using a quartz microbalance. In this case, the films were first incubated in a commercial solution containing simulated body fluids for at least 2 h. The sensors were then subjected to an aqueous solution containing 40 mg mL^−1^ albumin, and the changes in the weight over 2 h were determined. Both the morphology and the composition of such protective coating after deposition was determined by SEM and EDS elemental analysis. The sensitivity of the sensors was then determined by measuring the open circuit potential of the devices after 5 weeks of preconditioning using a solution of 0.1 M KCl.

## 3. Results and Discussion

### 3.1. Optimization of the Deposition Temperature and Roughness of the Sensitive Films

Theoretically, the performance of ISE is determined by the ion fluxes across the plasticized membrane as evidenced by multiple simulation studies [10,32,33]. These ion fluxes generate a diffusion layer, consisting of a change on the concentration of electrolytes near the surface of the electrode [34]. As such, these ion fluxes are significantly influenced by the morphology of the electrode surface and, more specifically, its roughness [35]. However, flat sensing films are normally used to simulate and describe ions sensors, without reflecting the effects of roughness and thickness that take place in real devices [10,36]. The thickness of the diffusion layer observed in ion-selective electrodes is typically in the range of 1–100 µm, which also lies within the same range as the roughness observed in ion sensors when they are deposited by drop casting [9,37]. This roughness has an impact on the diffusion layer, which is translated into a difference in the time response and stability of the devices [34,37]. In addition, such roughness can increase the interfacial resistance of the films due to a change on their capacitive behavior [38,39,40]. Consequently, the fabrication of highly smooth and homogeneous films is necessary to achieve a systematic study of the effects of thickness in the performance of the ion sensors.

**Figure 2:**
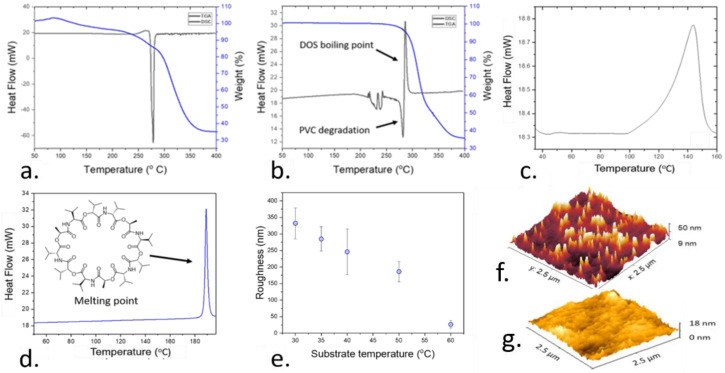
Results obtained during the thermal characterization of the sensitive ion films. (**a**). Combined thermogravimetric analysis (TGA, blue) and differential scanning calorimetry (DSC, black) plot of a pure PVC film, showing the thermal degradation of this polymer at high temperatures. (**b**). Combined TGA (blue) and DSC (black) plot of a plasticized polyvinyl chloride (PVC) film containing 2-(ethylhexyl) sebacate (DOS) as plasticizer. The previously observed degradation peak can be observed alongside an endothermal peak due to the boiling of DSC. (**c**). DSC plot of pure valinomycin, where the melting point reported at 190 °C can be observed. (**d**). DSC characterization of the valinomycin embedded inside a plasticized high molecular weight PVC film, showing a displacement on the endothermal peak at 140 °C. (**e**). Changes in the roughness of the sensing films as a function of the employed substrate temperature during the AACD process, showing an optimal temperature at 60 °C. (**f**). Atomic force microscopy (AFM) of a plasticized PVC film deposited under non-optimal conditions (70 °C) (**g**). AFM visualization of the surface of the sensing films deposited at 60 °C, showing the low film roughness.

Within the present work, AACD was adapted for the deposition of highly homogeneous films with controllable thicknesses. This deposition method was first optimized in terms of temperature and deposition time to achieve a fine control over the processing conditions and morphology of the films. The temperature of the deposition played a pivotal role in this process since it influenced the thickness, roughness, and stability of the films. While PVC is a thermoplastic, with high thermal resilience [41], the stability of the rest of the components of the films including the ionophore and the plasticizer at relatively high temperatures has not been established yet. To determine the optimal range for the temperature deposition, thermogravimetric analysis (TGA) and differential scanning calorimetry (DSC) was conducted on pure PVC (Figure 2a) and plasticized PVC films, after the incorporation of DOS plasticizer within a ratio of 1:3 (Figure 2b). These techniques allowed the determination of the minimum temperature at which the composition of the sensing films was compromised due to the boiling or thermal degradation of its components (PVC, or DOS).

The study of the thermal stability was additionally conducted on pure valinomycin, which is the ionophore employed in the current work. This ionophore is selective for the detection of potassium ions. However, the effects of high temperatures on the stability of ionophores such as valinomycin inside plasticized PVC films have not been reported yet. Early studies reported a conformational change of valinomycin molecule when embedded in lipid membranes at 40 °C [42]. Thus, this molecule is expected to be low at relatively high temperatures. The DSC thermogram of pure valinomycin (Figure 2c), and valinomycin when embedded inside the plasticized polymer film (Figure 2d) were measured to determine the temperature range for the deposition of the films.

After the thermal optimization of the fabrication conditions, the effects of the temperature on the roughness of the films was determined. Thus, the deposition temperature not only influenced the stability of the compounds incorporated inside the sensing films, but it also impacted the roughness of the films after deposition (Figure 2e). To corroborate these results, AFM imaging was conducted, allowing a mapping of the morphology of the films at a nanometer scale. This map could be used to compare the surface homogeneity of the polymer sensing films deposited under optimal and non-optimal conditions (Figure 2f,g).

When pure PVC was characterized by DSC, an exothermal reaction was observed at 303 °C which was followed by a loss of 65% of the weight determined by TGA. This process is a consequence of the pyrolysis of the polymeric chains of the PVC, which is catalyzed by a de-chlorination of the polymer chains, leading to a loss in the weight [43]. When the DOS plasticizer was integrated in the film, the previously observed exothermal reaction was followed by an endothermal peak at 275 °C. Such an endothermal reaction was attributed to the boiling point of DOS, which has been reported in the range of 256 °C [44]. In this case, the higher temperature at which this process took place inside the polymer film, in the range of 290 °C, was attributed to an interaction of the plasticizer with the polymer chains, which increased the stability of the plasticizer.

In the case of valinomycin, an endothermal peak was observed at 190 °C by DSC, which is consistent with the reported melting point of valinomycin. However, when such valinomycin was embedded inside a plasticized polymer film, an endothermal peak at 143 °C was recorded. When ionophores are embedded inside polymeric membranes, they are in a liquid-like state, where they can easily dispersed through the films [45]. As such, this peak was attributed to a conformational change of the valinomycin.

As determined by DSC and TGA, the polymer sensing films had to be deposited by a temperature lower than 140 °C to avoid the degradation of the components in the films. However, the deposition temperature also had an effect on the roughness of the films. When the deposition of the sensing films was conducted under different temperatures in the range of 30–60 °C, the film roughness decreased at higher temperatures, reaching a minimum at 60 °C with 27 ± 11 nm. Beyond this temperature, the roughness increased up to 424 ± 12 nm at 70 °C. As such, this temperature was employed within the following experiments for the fabrication of thin films.

### 3.2. Comparison of the Aerosol-Assisted Chemical Deposition (AACD) and Drop Casting Results for the Development of Homogeneous Sensing Films

To offer a better understanding of the film conditions for achieving an optimal homogeneity, different molecular weights of PVC were tested during the fabrication process of the sensing films. Up to three different molecular sizes of PVC were tested (49, 79 and 84 kDa). The roughness of the fabricated films was measured after depositing a film using AACD for 30 s at 60 °C. The obtained results were compared with the roughness obtained after depositing the films by the standard method based on drop-casting, using 100 µL and incubating the films overnight (Table 1).

Both the roughness and homogeneity of the sensing films became significantly lower after the use of AACD as the fabrication method. The lowest roughness achieved in this case was obtained when using high molecular weight PVC with 27 ± 11 nm, followed by medium molecular weight PVC with 92 ± 5 nm and, finally, low molecular weight PVC which showed the highest value with 122 ± 12 nm. On the contrary, when drop-casting was employed, the minimum roughness was achieved in the case of medium molecular weight PVC with 309 ± 153 nm, an order of magnitude higher than the roughness achieved by AACD. This roughness reached up to 943 ± 624 nm in the case of high molecular weight PVC. The high error rates obtained in the case of drop casting were additionally indicative of the poor reproducibility of this manufacturing method compared to AACD. Thus, the improvement of the sensing films in terms of roughness when using AACD as the fabrication method was demonstrated. Within the following experiments, sensing films fabricated by AACD at 60 °C using high molecular weight PVC were used.

### 3.3. Systematic Study of the Effects of Thickness on the Performance of the Plasticized Sensing Films

**Figure 3:**
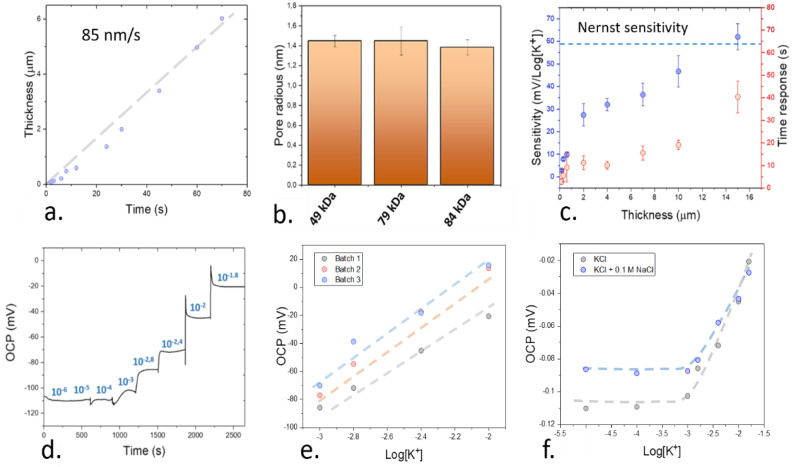
(**a**). Calibration of the film thickness using different deposition times by AACD. An increasing thickness was observed at higher deposition times as expected, allowing a fine control over the film dimensions. (**b**). Estimated pore size distribution of the low, medium and high molecular weight PVC films measured by Brunauer–Emmett–Teller (BET). (**c**) Changes in the sensitivity and response time of the sensing devices due to changes on the film thickness. The error bars represent the standard errors from the linear plot of the calibration. (**d**). Results obtained within a typical electrochemical characterization of an ion-selective electrode where the open circuit potential (OCP) of the working electrodes is monitored upon the use of different concentrations of electrolytes. (**e**) Testing of batch reproducibility by measuring the sensitivity of 3 different 15 µm thick sensors. (**f**) Determination of the selectivity against Na^+^ ions of the developed 15 µm thick sensing devices by the fixed interference method.

As determined in previous sections, the use of AACD can reduce the surface roughness of ion-selective electrodes down to the nanometer level. Such an improvement in the manufacturing conditions could be used for the fabrication of thin films with a thickness below the micrometer range (Figure 3a), which represented an improvement compared to the conventional drop-casting technique, where the typically achieved thicknesses are in the range of 100 µm [25].

One of the crucial parameters in ion-selective electrode films is the porosity of the employed materials. Such porosity is essential t allow the permeability of the ions inside the films. González-Bellavista et al. showed the superior performance of highly porous polymers such as polysulfone for the detection of nitrate ions in solution, with higher selectivity than the commercially available devices [46]. To study the influence of the polymer selection on the porosity of the films, BET was conducted. This method could be used to determine the average pore radius of the films deposited by AACD (Figure 3b). The pore radius of the plasticized PVC films remained within a similar range of 1.4 nm in all cases. As such, the changes in the molecular weight using AACD as the deposition technique were only observed to influence the roughness of the films, while keeping a similar porosity of the films.

To ensure a proper functionality of the sensing devices developed by AACD, the sensitivity of the ion sensors fabricated using plasticized high molecular weight PVC, and containing valinomycin as the ionophore and tetraphenyl borate was determined at different thicknesses (Figure 3c). This sensitivity was determined by exposing the ion sensors to different concentrations of potassium ions, and measuring the changes in the open circuit potential of the films (Figure 3d). The reproducibility of the sensors obtained by fabricating the films using AACD was additionally tested by characterizing 3 different electrodes produced within different batches. In all cases, the sensitivity of the devices was similar, with 62.4, 61.0 and 61.3 mV Log[K^+^]^−1^ respectively (Figure 3e). The limit of detection of the films was in the range of 10^−4^ M. This value was calculated by measuring the intersection between the calibration plot and the signal in a control sample with DI water with no ions. In addition, the sensors showed a good selectivity towards K^+^ ions when compared with Na^+^. This selectivity was evaluated by using the fixed interference method. In this case, a concentration of 0.1 M NaCl was added to the electrolyte solution, and the concentration of KCl was then increased (Figure 3f). The intersection between the standard calibration plot of the sensors with no NaCl and the mixed solution was then measured and used for the calculation of the selectivity. This selectivity was in the range of $k_{K^{+},\;Na^{+}}^{pot}$ = −1.8, which is similar to the reported selectivity coefficient of similar potassium-selective electrodes calculated by the same method ($k_{K^{+},\;Na^{+}}^{pot}$ = −2.1) [47].

An increase of 80 nm in thickness per second of deposition was obtained using the AACD technique for the deposition of high molecular weight PVC film. This deposition rate could be used for the fabrication of nanometric-size films, with up to 100 nm in thickness. When the devices were calibrated, the time-response increased upon the use of thicker films as expected due to the longer diffusion paths of these films. However, contrary to the established theory of ion-selective electrodes, a sub-Nernstian sensitivity of the ion-selective electrodes was observed at thicknesses below 15 µm. Only at 15 µm, a Nernst sensitivity in the range of 62 ± 6 mV Log[K^+^]^−1^ was obtained. Thus, these results suggest the presence of a different mechanisms of ion exchange that limits the performance of the ion sensing films at sub-micron scales that has not been reported yet. Such an ion exchange mechanism is related with the ion permeability of the sensing films at the nanometer scale. This change in the permeability properties of films at low thicknesses has been previously determined in the case of membranes for gas separation [48]. In this case, such changes in permeability were attributed to changes in phase combinations, appearance of metastable phases and formation of domains with higher stresses in the films, which could change the structure of the films [49]. In the present case of ion sensing films, to determine the possible causes of this changes in the permeability properties of the polymeric films, a further morphological study of the films was conducted.

### 3.4. Systematic Study of the Effects of Thickness on the Performance of the Plasticized Sensing Films

**Figure 4:**
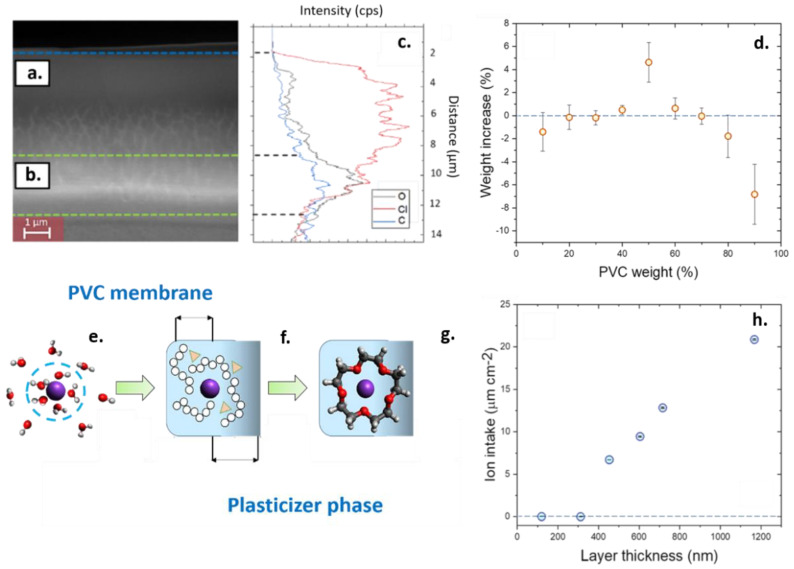
(**a**). Scanning electron microscopy (SEM) imaging of the plasticized polymer films, evidencing a non-homogeneous distribution of the plasticizer and the PVC. The superficial zone in direct contact to the aqueous sample showed a higher presence of PVC chains and a low plasticization. (**b**). Within the bulk phase of the ion sensing films, there was a higher presence of plasticizer and striations, indicative of the porosity of the films. (**c**). Energy-dispersive X-ray spectroscopy (EDS) linear scan of the composition of the sensing films, indicating the relative presence of oxygen, carbon and chlorine. (**d**). Quartz microbalance study of the absorption of water in the PVC films containing different amount of plasticizers. (**e**). Schematic representation of the three-step recognition process. The ions in solution are surrounded by a water shell, increasing their size. (**f**). the first step in the sensing process is the diffusion of the ions inside the membrane through the spacing created between the polymers and plasticizer. (**g**). The complexation with the ionophore process takes place. (**h**). Ion absorption properties of polymeric membranes at different thicknesses. The error bars represents 5 times the standard deviation of 1000 data points of the quartz microbalance measurements, and were in the range of 5% of the signal measurement.

To study the causes behind the observed changes in sensitivity of the sensing devices, the cross-section of the plasticized PVC films was observed using SEM (Figure 4a). Such cross-section was obtained by freezing the sensing films deposited by AACD using liquid nitrogen to preserve their morphology and sectioning them. This experiment revealed two clearly distinctive areas on the films; a dense upper layer and a porous bulk phase (Figure 4b). The composition of both areas could be determined by using EDS linear scanning (Figure 4c). Such linear scanning showed for the first time a non-homogeneous distribution of the DOS plasticizer and PVC across the sensing film. This non-homogeneous distribution was consistent with the changes observed in the sensitivity of the devices at extremely low thicknesses since the permeability of sensing films is highly dependent on the composition in terms of plasticizer:polymer ratio.

The differences on the permeability of the sensing films as a function of the amount of plasticizer incorporates in the film were determined by quartz microbalance (Figure 4d). An optimal water intake was observed at 50 wt.%. This result is consistent with the previous literature, which states a value around 40 wt.%. as an optimal sensor composition membrane [50]. At this concentration of plasticizer, the presence of water inside the films facilitates the movement of ions between the sensor and sample phases [51]. An increase in the weight of the films after the exposure to DI water of 4.6 ± 1.7 wt.% was measured in the case of 50 wt.% of DOS, being consistent with previous reported work using nuclear magnetic resonance (NMR), that indicate a presence of 2 wt.% of water inside 60 wt.% PVC plasticized films after equilibration overnight [51]. 

The presence of water molecules inside the sensing film was necessary to ensure the sensing since the ion recognition process inside ion sensors requires that the ions in solution diffuse inside the polymeric films [52]. This change has an energy loss associated due to the displacement from the water shell. However, one of the limiting steps is linked to the diffusion of the electrolytes inside the polymer films (Figure 4e). In the case of the ion sensors at low thicknesses, the compositional changes in the film could act as a diffusional barrier that prevents the intake of ions inside the films. Such compositional changes could cause a fast degradation of the films since low-plasticized films, with a content of plasticizer in the range of 10 wt.%, were observed to release PVC microparticles in solution, accelerating the film degradation. In addition, DOS-rich films can leach the plasticizer onto the aqueous sample (Figure S1). Thus, when the thickness of the films is low, given the small ratio of bulk to the surface area, the diffusion of plasticizer and ionophores onto the sample is accelerated, which decreases the sensitivity of the devices. This decrease in the sensitivity due to the leaching of plasticizer and ionophores has been observed in previous studies focused on the stability of the PVC-based ion sensors [17].

The leaching of DOS plasticizer in the water samples was consistent with the reduced sensitivity observed in these devices at thicknesses below 15 µm. However, the study of the device sensitivities alone does not provide enough information about the diffusion of ions inside these films. In some cases, a low sensitivity can also be a consequence of a co-diffusion inside the films of the target cation and the counter ion [11,53,54]. Thus, a monitoring of the mass changes inside the films at sub-micron scales was conducted using quartz microbalance to determine how the thickness of the films influenced the absorption properties of the sensing films (Figure 4h).

The EDS elemental analysis revealed a high concentration of PVC chains at the surface of the plasticized polymer film after pre-conditioning the devices in solution for 12 h. Such higher concentration of PVC chains was reflected on a higher intensity of the Cl elements in the upper layer of the films. On the contrary, a higher intensity of oxygen and carbon elements was observed at the region close to the substrate, indicating an elevated concentration of plasticizer within the bulk of the films, since the PVC chains do not contain oxygen atoms. As such, the plasticizer contributed to the creation of striations in the film as can be observed by SEM, which could favor the diffusion of ions. The presence of such plasticized regions with striations can preserve the sensitivity of the ions sensors since the presence of plasticizers is essential for the ion and ionophore mobility [55] as well as the diffusion of ions [56]. This fact becomes crucial in the ion sensors since the diffusion coefficient of ions inside the polymeric films is significantly lower than in water samples [57]. Thus, only when an optimal concentration of 50 wt.% plasticizer inside the PVC films was employed, a good absorption of water was obtained. 

When the ion intake of the plasticized polymer films was tested at different thicknesses, a linear increase in the intake of potassium ions was obtained within the 500–1200 nm, and a negligible ion intake was also recorded below 150 nm. These results were consistent with the observed decrease in the sensitivity obtained when measuring the electrochemical performance of the sensing films, and indicated a low diffusion of ions at sub-micron scales. The causes of this loss in sensitivity of the ion films were attributed to the non-homogeneous distribution of polymer and plasticizers, which limited the intake of ions by the sensing films. Such non-homogeneous distribution of polymer and plasticizer also promoted the degradation of the films through the leaching and material desorption.

### 3.5. Development of a Biocompatible Protective Coating for the Thin Films

As determined in previous sections, one of the limiting factors regarding the stability of ion-selective electrodes is the degradation of films due to the migration of polymers and plasticizers into the sample solution. Such migration not only reduces the sensitivity of the devices, but also it can compromise the biocompatibility of the devices due to the leaching of plasticizers [58,59]. To avoid this effect, multiple approaches such as gamma irradiation [58], and the use of protective coatings containing elastomers [60] have been developed. In particular, the use of an organosilicon upper layer to suppress the degradation of the ion-selective electrodes shows promise for the development of low-cost and highly reliable devices that can operate for months with no effects on the sensitivity. This concept was proven by Kumar Joon et al. [60] who drop-casted elastomers onto the ion-selective electrodes based on plasticized PVC. Such protective film not only improved the reproducibility of the potentiometric signals over time, but it could also preserve the sensitivity after 8 days of continuous pre-conditioning in a 0.1 M KCl solution. As such, this approach represented a promising alternative for the development of highly stable ion sensors for biomedical applications and implanted devices.

**Figure 5:**
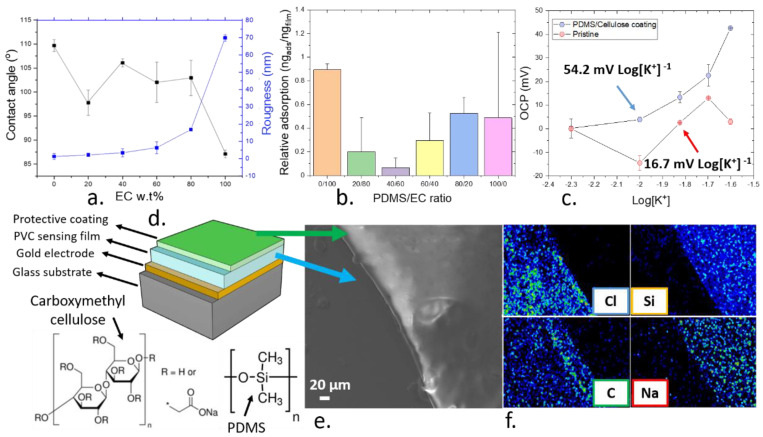
(**a**). Relationship between the water contact angle and the roughness of the poly(dimethyl) siloxane (PDMS)/ethyl cellulose (EC) films at different compositions of EC. (**b**). Characterization of protein adsorption of the ion films by quartz microbalance when using a solution containing 40 mg mL^−1^ albumin on the PVC films coated with different PDMS/EC protective layers. (**c**). Sensitivity of the pristine (red) and coated (blue) ion sensors after 5 weeks of continuous measurement in the presence of 0.1 M KCl. (**d**). Schematic representation of the ion sensors containing a substrate, contact electrode, PVC sensing film and protective coating. (**e**). SEM imaging of the PVC sensing film (left) and the PDMS/EC protective coating (right). (**f**). EDS elemental mapping of both the plasticized PVC film and the PDMS/EC protective coating, confirming the composition of the films.

Within the present work, a mixed matrix containing PDMS elastomer and EC was developed as an upper protective film to enhance the performance of ion sensors. The combination of both PDMS and EC could prevent the chemical release and reduce the protein fouling effects when the sensors were exposed to a simulated biological environment. Firstly, the ion sensing films were deposited by using AACD under optimized conditions. An upper layer of both PDMS and EC using the same processing conditions was then applied. The hydrophobicity and the roughness of such films were characterized to establish the suitability of AACD as the fabrication method for such protective coating (Figure 5a,b). The surface hydrophobicity of films plays a pivotal role in the adsorption of biological components onto the devices, since the aggregation of proteins onto surfaces is highly driven by hydrophobic interactions [61]. In addition, the water permeability of elastomers tends to be low, which could limit the diffusion of ions and reduce the sensitivity of the sensing films. Consequently, the protective coating also incorporated a cellulose derivative to tailor the hydrophobicity and permeability of the ion sensors. Thus, smooth and hydrophilic surfaces are desired to improve the sensing performance of the films containing the protective coating. After the deposition of the protective coating, as the proportion of PDMS compared to EC increased, the film became more hydrophobic up to a contact angle of 109 ± 1° when pure PDMS was employed. By contrast, the roughness of the films increased upon the use of higher concentrations of EC. An optimal composition to avoid the adsorption of albumin was then found when the films consisted of 40 wt.% PDMS and 60 wt.% EC.

To demonstrate the superiority of this protective coating in the prevention of protein fouling, a quartz microbalance characterization was conducted. This method could be used to calculate the increase in the weight of the polymer films containing the protective coating after the exposure to albumin, which was correlated to the adsorption of such proteins. When optimal films containing 40 wt.% PDMS and 60 wt.% EC were employed, an adsorption of albumin of 0.07 ± 0.79 ng_alb_ ng_film_^−1^ was observed when the sensors were exposed to an aqueous solution containing 40 mg mL^−1^ albumin. This concentration of albumin was chosen since it is in the range of the concentration typically found in normal patients [62]. On the contrary, a maximum adsorption was reported when pure EC was employed as the protective film, with 0.90 ± 0.05 ng_alb_ ng_film_^−1^. Due to the lowest adsorption of albumin at this concentration, such composition was used to study the improvement of the stability of the PVC-based ion sensors over time.

When a protective layer containing 40 wt.% PDMS and 60 wt.% EC was deposited onto the standard PVC-based ion sensors by AACD, a Nernst sensitivity of the polymer sensors was obtained. Such sensitivity remained stable for at least 5 weeks after their fabrication, with 54.3 ± 16.7 mV Log[K^+^]^−1^. By contrast, the sensitivity of the pristine PVC-based ion sensors with no protective layer decreased to a value of 16 ± 18 mV Log[K^+^]^−1^ (Figure 5c) after 5 weeks. This result confirms the beneficial effect of the use of protective layers to improve the stability of ion sensors over time, allowing a reliable long-term monitoring of ions. The morphology and composition of this upper protective film, based on PDMS and EC onto a 10 µm thick PVC film (Figure 5d) was confirmed by EDS. In this case, the protective layer containing 40 wt.% PDMS and 60 wt.% E was deposited onto a plasticized PVC film, and a side of the film was covered during the deposition to prevent it from being coated with the protective film. This experiment allowed the observation of both the PVC and PDMS/EC films (Figure 5e). Such film was shown to be smooth, as expected by the results from the profilometer. In addition, the expected composition of both films was observed. In the case of PVC both Cl and C elements were detected by the EDS, while C and Si were observed on the protective coating side.

Thus, AACD was proven to be a useful fabrication technique for the development of highly stable and homogeneous ion sensors. The versatility of such a technique could also be applied for the deposition of protective films, improving the stability of such ion sensors.

## 4. Conclusions

This work reports for the first time the incorporation of aerosol-assisted chemical deposition for the fabrication of highly homogeneous and smooth polymeric sensing films with controllable thicknesses (in the nanometer range). This technique was optimized for the deposition of plasticized PVC films, allowing the fabrication of thin sensing films with a low roughness, in the range of 27 nm. The high homogeneity levels achieved by this technique allowed the objective study of the effects of thickness on the ionic diffusion of films.

We applied this AACD method for the fabrication of miniaturized potassium-selective films with different thicknesses. The effects of the film size on the performance of standard PVC-based ion-selective electrodes were studied by determining the sensitivity and time-response of the potassium-selective devices. Contrary to the classical theory based on the Nernst equations, a dependency on the polymeric thickness with the absorptive capabilities of the films was found, which ultimately reduced their sensitivity when working on a sub-micron scale but reducing the time-response of the devices. A physical-chemical characterization of the sensing films deposited by AACD was additionally conducted by quartz microbalance and SEM on these films to determine the cause of this sensitivity loss. A bias in the composition near the superficial layers of sensor was found, which lead to a low absorption of ions by the films at nanometric scales. The study with quartz microbalance allowed the determination of the contributions from the mass absorption of ions in the sensitivity of the devices at nanoscale, demonstrating this decrease in the ability of the films to retain ions. To improve the lifespan of the polymeric ion sensors, an upper protective layer based on PDMS and EC was designed. This protective coating was deposited onto the potassium-selective sensing films, and showed a low protein adsorption while maintaining a Nernst sensitivity of the devices. The composition of this protective film was first optimized in terms of hydrophobicity and surface roughness, achieving a superior performance when 40 wt.% PDMS and 60 wt.% EC was employed. By implementing this protective film, the sensitivity of the sensors remained stable up to 5 weeks of continuous preconditioning inside a highly concentrated solution of 0.1 M KCl. This result represented an improvement with respect to the standard approach based on plasticized PVC alone. Thus, AACD has been demonstrated to be an optimal non-vacuum and low-cost technique for the fabrication of ion sensors, with well-controlled dimensions, with a plethora of potential applications in the mass manufacturing of ion sensors that can be used in healthcare, agriculture or water quality assessment, among others.

## Figures and Tables

**Table 1:** Arithmetical mean roughness of the plasticized PVC films deposited onto gold electrodes by AACD and drop casting. Low, medium and high molecular weight PVC were deposited for 30 s under optimal conditions.

Sample	AACD Roughness (nm)	Drop Casting Roughness (nm)
High molecular weight PVC	26.5 ± 11.1	943 ± 624
Medium molecular weight PVC	92.3 ± 5.3	309 ± 153
Low molecular weight PVC	122 ± 17	458 ± 216

## Data Availability

Not applicable.

## Supplementary Materials

The following are available online at https://www.mdpi.com/article/10.3390/nano11040938/s1, Figure S1: (a). Comparison of the FTIR spectrum of DOS plasticizer (red) and the leach exudate collected from a pure water sample after exposure to a 90% wt.% plasticised PVC film (black). The observed peaks were consistent with the alkyl (1950–2850 cm^−1^) and ester (1730 cm^−1^) stretches. (b). Molecular structure of bis-(2-ethylhexyl) sebacate. (c). SEM visualisation of a PVC microparticle (1.2 µm wide) obtained from low-plasticized sensing film. The composition of such microparticle was determined by EDS, with a high content of (d) C and (e) Cl elements.
