# Supplementary material for: Integration of an Aerosol-Assisted Deposition Technique for the Deposition of Functional Biomaterials Applied to the Fabrication of Miniaturised Ion Sensors
Source: Nanomaterials (Basel). 2021 Apr 7;11(4):938. doi: 10.3390/nano11040938 (PMC8067610; doi:10.3390/nano11040938)
Supplement: Supplementary file 1 [file nanomaterials-11-00938-s001.pdf]

Supplementary Material

Integration of an Aerosol-Assisted Deposition Technique for the Deposition of Functional Biomaterials Applied to the Fabrication of Miniaturised Ion Sensors

Antonio Ruiz-Gonzalez and Kwang-Leong Choy *

Institute for Materials Discovery, Faculty of Mathematical & Physical Sciences, University College London, 107 Roberts Building, Malet Place, London WC1E 7JE, UK; a.gonzalez.16@ucl.ac.uk
* Correspondence: k.choy@ucl.ac.uk

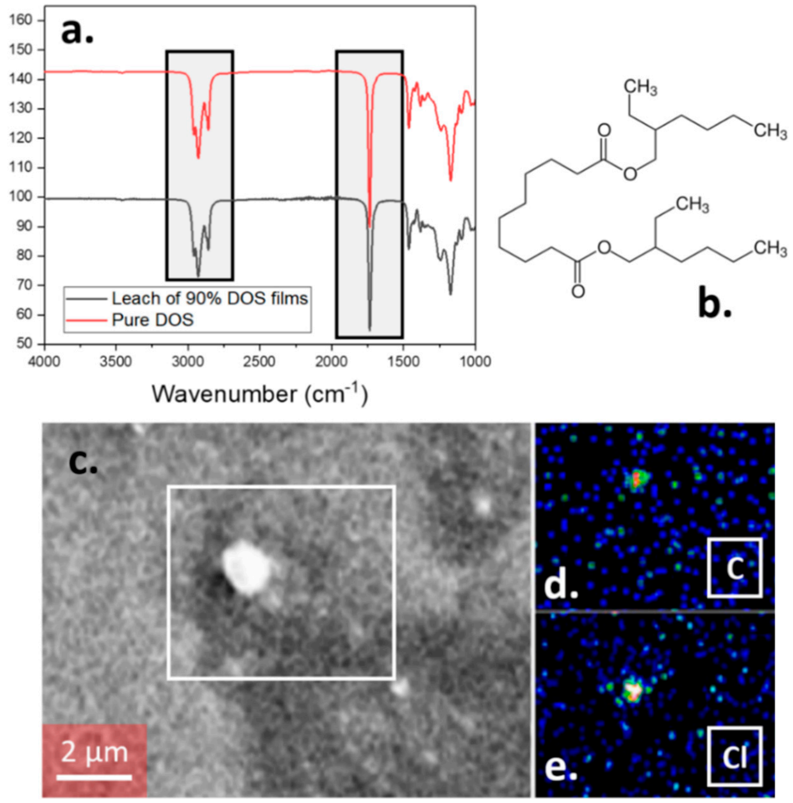

Figure S1. (**a**). Comparison of the FTIR spectrum of DOS plasticizer (red) and the leach exudate collected from a pure water sample after exposure to a 90% w.t% plasticised PVC film (black). The observed peaks were consistent with the alkyl (1950–2850 cm^{-1}) and ester (1730 cm^{-1}) stretches. (**b**). Molecular structure of bis-(2-ethylhexyl) sebacate. (**c**). SEM visualisation of a PVC microparticle (1.2 µm wide) obtained from low-plasticized sensing film. The composition of such microparticle was determined by EDS, with a high content of (**d**) C and (**e**) Cl elements.
